# Molecular and pathophysiological intersections between arterial hypertension, pulmonary inflammation and diabetes mellitus

**DOI:** 10.3389/fphar.2025.1734861

**Published:** 2026-04-01

**Authors:** Higo José Neri da Silva, Antônio Carlos Melo Lima-Filho, Ester Miranda Pereira, Ian Jhemes Oliveira Sousa, Francisco Valmor Macedo Cunha, Massimo Lucarini, Alessandra Durazzo, Leonardo da Rocha Sousa, Daniel Dias Rufino Arcanjo

**Affiliations:** 1 LAFMOL–Laboratory of Functional and Molecular Studies in Physiopharmacology, Department of Biophysics and Physiology, Federal University of Piauí (UFPI), Teresina, Piauí, Brazil; 2 RENORBIO–Northeast Biotechnology Network, Federal University of Piauí (UFPI), Teresina, Piauí, Brazil; 3 CEUMA University (UNICEUMA), Imperatriz, Maranhão, Brazil; 4 Laboratory of Immunogenetics and Molecular Biology (LIB), Federal University of Piauí (UFPI), Teresina, Piauí, Brazil; 5 Faculty of Medicine, UNINOVAFAPI University Center, Teresina, Piauí, Brazil; 6 CREA-Research Centre for Food and Nutrition, Rome, Italy; 7 Department of Information, Environment, Health and Food Production, Federal Institute of Education, Science and Technology of Piauí (IFPI), Teresina, Piauí, Brazil

**Keywords:** inflammation, inflammatory mediators, NF-kappa B, oxidative stress, tumor necrosis factor alpha

## Abstract

The pathophysiological interactions between arterial hypertension (AH), pulmonary inflammation (PI) and diabetes mellitus (DM) have been widely studied due to their clinical relevance and global impact. Chronic inflammation and oxidative stress act as central axes that exacerbate these conditions, creating a pro-inflammatory and harmful environment. This study aims at characterizing the immunological and molecular mechanisms shared by these pathologies, highlighting the signaling pathways that connect these conditions and their implications for therapeutic management. The scoping review followed JBI and PRISMA-ScR guidelines. Descriptors such as “Hypertension”, “Pulmonary inflammation” and “Diabetes” were used, and then 48 articles were selected. Data extracted included inflammatory markers, molecular mediators and pathogenic mechanisms. The methodology ensured the consistency of the analysis and relevance of the findings of articles published in the last 10 years. The results indicated that arterial hypertension aggravated pulmonary inflammation by systemic inflammatory responses, promoted the activation of NF-κB and increased pro-inflammatory cytokines, such as TNF-α, compromising pulmonary vascular integrity and favoring tissue remodeling. Simultaneously, chronic hyperglycemia in diabetes mellitus intensifies these processes by inducing oxidative stress and endothelial dysfunction, reducing the bioavailability of nitric oxide (NO). These effects are possibly potentiated by the activation of the renin-angiotensin-aldosterone system and the mineralocorticoid receptor, promoting vasoconstriction and inflammation. Hyperglycemia stimulates the production of reactive oxygen species (ROS) and activates NF-κB, leading to the release of TNF-α and IL-6, perpetuating vascular dysfunction. Furthermore, macrophage- and neutrophil-mediated IP contributes to pulmonary vascular remodeling and impaired gas exchange, aggravating hypertension and DM. Interventions such as SGLT2 inhibitors have demonstrated potential to modulate the three affected systems, while biomarkers such as NF-κB and TNF-α emerge as promising targets for integrated strategies, since they are considered key modulators of the conditions under study. Indeed the intersection between these conditions could be mediated by shared pathways, as mentioned above, which maintain systemic and local inflammation. These markers play crucial roles in the progression of the analyzed conditions, highlighting the need for integrated therapeutic strategies modulating inflammation, oxidative stress, and vascular remodeling. Those approaches may mitigate the interdependent impacts of these diseases and offer new avenues for personalized treatments.

## Graphical Abstract

**Figure:**
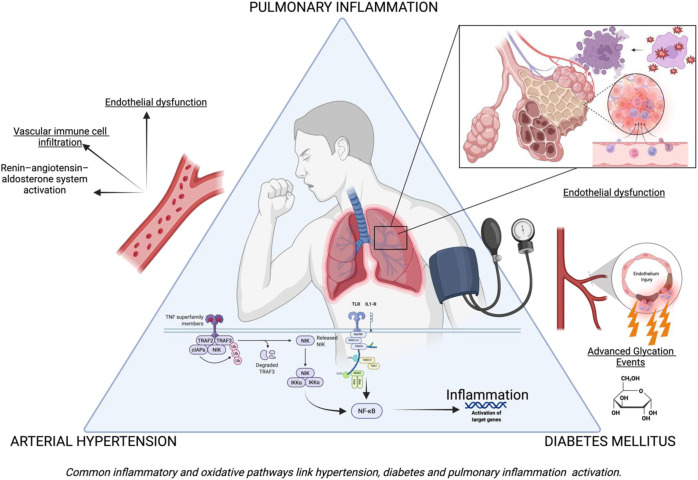

## 1 Introduction

The inflammatory process is considered a defense mechanism of the human body generated against harmful agents or infections. The cells residing in the different access routes to the human body, such as alveolar macrophages, dendritic cells and epithelial cells, play fundamental roles in the detection and initial response to these stimuli, releasing chemical mediators, such as interleukins and growth factors, to recruit neutrophils and lymphocytes to the injured site (Aghasafari et al., 2019).

Loss of function as result of a chronic inflammatory process suggests a potential interaction on irreversible tissue damage, capable of causing changes in the tissue microenvironment (Barberà, 2013). With more than 545 million people affected worldwide (GBD 2019 Tobacco Collaborators, 2021), chronic inflammatory lung diseases such as asthma and chronic obstructive pulmonary disease (COPD) contribute significantly to global mortality, with a direct impact on the quality of life of individuals and on the economy of health systems (Labaki and Han, 2020).

In asthma, persistent inflammation results in bronchial hyperreactivity and remodeling of lung structures, while in COPD there is progressive destruction of lung tissue and airway obstruction (Blanner et al., 2007; Aghasafari et al., 2019). The pre-existence of comorbidities such as arterial hypertension (AH), diabetes mellitus (DM), cardiovascular diseases (CVDs) and obesity, has been identified as determining factors for an increase in this response and worsening of clinical conditions (Khanna et al., 2022). In particular, both asthma and COPD are associated with significant hypertension, as demonstrated in several studies (Mannino et al., 2008; Ferguson et al., 2014; Mahishale et al., 2015; Christiansen et al., 2016; GBD Chronic Respiratory Disease Collaborators, 2020).

Hypertension is a multifactorial clinical condition characterized by a persistent elevation of blood pressure equal to or above 140 and/or 90 mmHg. Its risk factors include advanced age, hypercholesterolemia, obesity, diabetes mellitus, morphological changes in target organs, excessive consumption of ultra-processed foods, and a sedentary lifestyle (Brazilian Society of Cardiology SBC, 2017; Barroso et al., 2021; Mills et al., 2020; Rezende et al., 2024). According to the World Health Organization (WHO), approximately 1.4 billion people have hypertension, but only approximately 200 million have it under control (WHO, 2021). In Brazil, approximately 25% of adults and more than 60% of the population over 60 years of age are affected by hypertension (Brazilian Society of Cardiology SBC, 2017).

At first glance, pulmonary inflammation (PI), arterial hypertension, and diabetes may seem to belong to distinct pathophysiological domains. However, scientific evidences suggest a deep association between them (Simpson et al., 2020; Tene, Robalino, and Pedreáñez, 2023). Chronic inflammation and oxidative stress generated by hypertension and PI are common bridges that also play critical roles in diabetes (Hertiš Petek et al., 2022; Zhang et al., 2023). The existence of pathways involving shared intracellular cascades between these conditions increases the possibility of studies and designs of new therapeutic strategies. Therefore, this scoping review a paper aims at characterizing the immunological/molecular and pathophysiological mechanisms shared between arterial hypertension, pulmonary inflammation, and diabetes mellitus, with the purpose of highlighting the interdependent connections that can aggravate these chronic conditions and identify potential therapeutic targets for integrated treatments.

## 2 Methodological aspects

This study is a scoping review prepared according to the six methodological steps recommended by the JBI: determination of the research question; identification of relevant studies; selection of articles; data extraction; separation, summarization and reporting of results; and dissemination of results.

The Preferred Reporting Items for Systematic Reviews and Meta-Analyses for Scoping Reviews (PRISMA-ScR) checklist was also used to guide the construction of this review. The elaboration of the guiding research question was based on the PCC mnemonic strategy (population, concept and context), in which “P” was given to individuals with arterial hypertension (AH), “C” to molecular mechanisms and inflammatory markers in AH, IP and DM and “C” to studies that elucidate the pathophysiological mechanisms and inflammatory mediators in AH, IP and DM. The research question that arose was the following: How do molecular mechanisms and inflammatory markers mediate the multidirectional relationship between arterial hypertension, pulmonary inflammation and diabetes mellitus, affecting the pathogenesis and progression of the aforementioned conditions?

To identify relevant studies, searches were conducted from February to November 2024 by two independent reviewers in the MEDLINE databases, PubMed, Academic Google, Web of Science, EMBASE, and ScienceDirect. The search strategy in the databases was carried out in three stages. In the first, an initial limited search in Academic Google was performed using the descriptors related to the PCC mnemonic: “Hypertension”, “Pulmonary inflammation”, “Lung disease”, “Hypertension pulmonary”, “Chronic respiratory diseases”, “Diabetes and hypertension”, “Diabetes and pulmonary inflammation” and “Diabetes and lung disease”, standardized and indexed in the MeSH (Medical Subject Headings) vocabulary, in order to verify words present in the title and abstract of the articles and relevant indexing terms. In the second stage, the other databases were considered and the identified words and indexing terms were associated with the descriptors through the Boolean operators AND and OR, resulting in different strategies in each database (Supplementary Table S1). In the third stage, the reference list of all studies included in the review was analyzed.

For the selection process, all studies found were transferred to the EndNote software (http://www.endnote.com/), and duplicates were excluded. The selection was then carried out in two stages: in the first, the titles and abstracts were read, and in the second, the full text was read. After reading the titles and abstracts, the studies that met the inclusion criteria were selected for the next stage. Those in which it was not possible, by reading only the title and abstract, to identify whether there was determination of inflammatory mediators and molecular markers related to HA, IP and DM, were excluded. After reading the full texts, the studies that analyzed the aforementioned markers and mediators were selected. The selection process was carried out by two independent reviewers, with the help of the Rayyan QCR application (https://www.rayyan.ai/). Disagreements were resolved with a third reviewer.

Following methodological guidance for scoping reviews, the analysis emphasized the mapping of available evidence and the description of key concepts and study characteristics, without prioritizing critical appraisal of individual studies, given the heterogeneity of the included designs.

Data extraction was guided by a form developed jointly by the authors, which included/categorized the following data: title, author, year, journal and key information. The extracted data were presented in tables, accompanied by a narrative caption. Further details of the search can be found in the Supplementary Material.

## 3 Results and discussion

**FIGURE 1:**
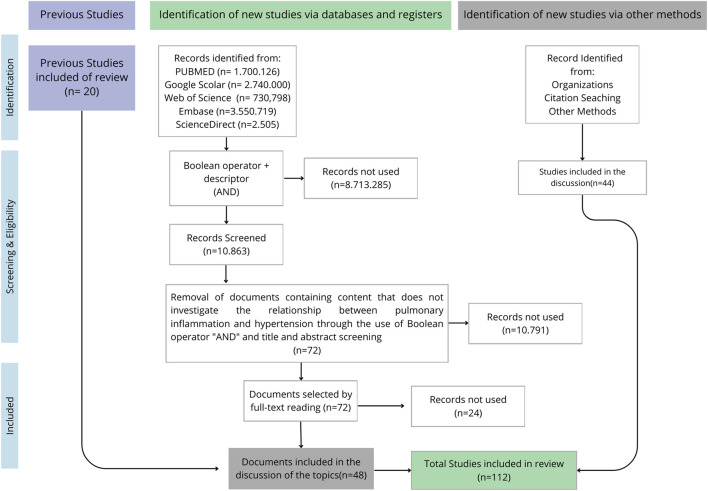
Flowchart of the protocol for identification and selection of scientific articles.

After applying the search descriptors to the databases listed in Supplementary Table S1, approximately 11,000 articles were identified. As shown in Figure 1, the first selection was based on the title and abstract of the articles. Excluding studies focused on pathologies and systems other than the respiratory system, a total of 72 articles were found, and the full text was read. Of these, 24 were excluded because they did not answer the research question, resulting in 48 articles. Of these, 22 studies were used to construct Supplementary Table S2, on the relationship between arterial hypertension and pulmonary inflammation, while 26 were used to construct Supplementary Table S3, which addresses the intersection between arterial hypertension, pulmonary inflammation and diabetes. The evidence presented in the studies selected for Supplementary Tables S2, S3 supported the writing of topics 3.1, 3.2 and 3.3.

### 3.1 Pathophysiological mechanisms associated between arterial hypertension and pulmonary inflammation

Hypertension is understood to be the most important risk factor for the development of CVDs according to modern lifestyle habits. Together with other dangerous blood pressure alterations, this condition is responsible for approximately 8.5 million deaths from stroke, ischemic heart disease, kidney and vascular diseases worldwide (Oparil et al., 2018). According to the World Health Organization (WHO), approximately 1.4 billion people have hypertension, but only about 200 million have it under control (WHO, 2021).

Thus, when left uncontrolled, there is a greater risk of acute myocardial infarction, coronary artery disease, angina, thrombosis and cerebral hemorrhage, in addition to kidney damage. Worldwide, between 2006 and 2015, hypertension caused losses in productivity and income estimated at 4.18 billion dollars. In Brazil, there are approximately 36 million patients with hypertension, most of whom are elderly (Brazilian Society of Cardiology SBC, 2017). Typically, these conditions are treated with diuretic drugs, angiotensin-converting enzyme (ACE) inhibitors and calcium channel blockers, which are capable of reducing mortality and harmful effects on the cardiovascular system and other organs (WHO, 2021).

HA induces a systemic inflammatory response, promoting the release of pro-inflammatory cytokines and elevation of leukocyte infiltration, the impact of which reaches other systems in addition to the cardiovascular system, such as the respiratory system due to its functional proximity and physiological interdependence (Supplementary Table S2) (Oparil et al., 2018).

Pulmonary inflammation is characterized by the immune system’s response to infectious agents, irritating particles (dust and/or toxic gases), or tissue damage. The release of inflammatory mediators, such as cytokines, chemokines, and growth factors, regulates a complex cascade of cellular events involving immune system cells, fibroblasts, and local endothelial cells (Mikołajczyk et al., 2020). Several factors, such as continuous exposure to harmful agents and/or tobacco consumption, can contribute to the progression of immune activation or interruption of pulmonary inflammation in cases of smoking cessation (Aghasafari et al., 2019). According to 2019 data from the Global Burden of Disease (GBD), approximately 1.14 billion people are smokers, of which 155 million are between 15 and 24 years of age, with deaths estimated at 7.69 million. Between 1990 and 2019, more than 200 million deaths were caused by smoking, totaling losses of more than 1 trillion dollars worldwide (GBD, 2019 Tobacco Collaborators, 2021).

Another example of respiratory inflammation is acute pneumonia, which affects the alveoli and bronchi of the lungs. It is divided according to where the infection was acquired, which can be in the community or in a hospital. In both cases, the participation of *Streptococcus pneumoniae*, *Staphylococcus aureus*, *Mycoplasma pneumoniae*, and some viruses, such as influenza, respiratory syncytial virus, coronavirus, and adenovirus, stands out (Torres et al., 2021). Even in the face of the primary barriers of the immune system, such as immune factors associated with the mucosa, ciliary beat, mucus production, mucin release, and small diameter of the bronchial tree, these pathogens develop in the lung parenchyma and induce the release of pro-inflammatory cytokines after activation of receptors for pathogenic molecular patterns. In a complex cascade of reactions, Toll-like receptor (TLR) activation of innate immune cells occurs, such as alveolar macrophages and innate lymphoid cells (B1a, CLI-1, CLI-2 and CLI-3), followed by the migration of transcription factors such as activator protein 1 (AP-1) and nuclear factor к-B to the nucleus (NF-кB). Surfactant proteins (SP-A and SP-D) and interleukins (IL) are then released into the alveolar environment, inducing the activation of more immune cells and the recruitment of neutrophils, B lymphocytes and helper and cytotoxic T lymphocytes. Many pneumocytes undergo apoptosis, resulting in increased inflammation and changes in surfactant fluid production. Finally, resident memory cells are formed and wait in the lymphoid areas of the airways for a new infection (Snyder and Farber, 2019; Zhao et al., 2024).

In addition to leukocytes and resident lung cells, platelets also play an important role in protecting/modulating against microorganisms, either by assisting in transporting neutrophils to the lung tissue region or by associating with bacteria and leukocytes, so that their low levels are associated with worsening inflammation in animal models (Stoppelaar et al., 2014). This association is exemplified by the activation of platelet-activated factor (PAF) and protease-activated receptor 2 (PAR2), which plays a key role in the development of the pathophysiology of inflammatory lung diseases. The aforementioned biomarkers participate in the immune response after neutrophil activation in the alveolar environment, increasing the local inflammatory response. Blocking the action and interaction of PAF and PAR2 in *in vitro* and *in vivo* experiments demonstrates that there is a reduction in NF-кB activation and intracellular calcium signaling, significantly reducing lung damage (Silva et al., 2023).

**FIGURE 2:**
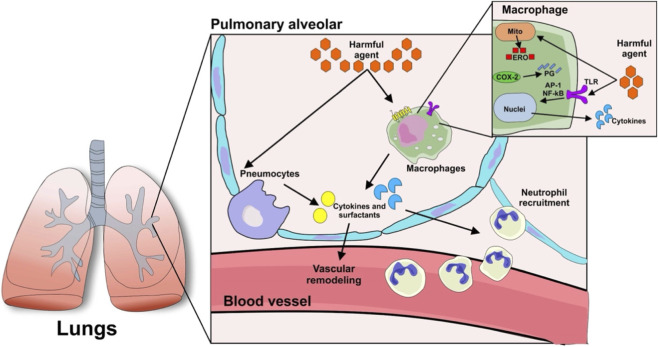
Molecular Mechanisms of Pulmonary Inflammation. After contact with a harmful agent within the alveolar environment, there is activation of leukocytes such as resident macrophages via Toll-like receptor (TLR), with subsequent activation of transcription factors (AP-1, NF-кB) and release of pro-inflammatory mediators (cytokines, prostaglandins, ROS). Neutrophil recruitment and pneumocyte activation produce an inflammatory microenvironment that progressively induces pulmonary vascular remodeling.

Demonstrating the participation of pathways other than prostaglandins, ROS, NO and metalloproteinases in the pathophysiology of pulmonary inflammation, experimental treatment of pulmonary arterial hypertension (PAH) with lysosomal autophagy inhibitors ROC-325, chloroquine and hydroxychloroquine reduce vascular remodeling and vasoconstriction resulting from local inflammation (Jones et al., 2019; Bao et al., 2023). Thus, as shown in Figure 2, several factors and figurative elements influence the development of the pulmonary inflammatory process.

Thus, within each leukocyte and cell exposed to the pulmonary inflammatory environment, prostaglandins are produced by the action of cyclooxygenase 2 (COX-2), modulating local inflammatory and vascular activity. As an example, prostaglandin H2 (PGH2) is a precursor of vasodilatory prostaglandins D (PGD2) and prostacyclin (PGI2), in addition to vasoconstrictor prostaglandins E (PGE2), F (PGF2) and tramboxane (TX). Such vasodilatory actions, via protein Gs and increased kinase A (PKA) activity, or vasoconstrictor actions, via protein Gq with production of inositol triphosphate (IP3) and release of intracellular calcium, regulate the activity of pulmonary blood vessels (Narumiya et al., 1999; Ciumărnean et al., 2020).

In adults, PAH is characterized by increased pulmonary vascular remodeling, associated with vasoconstriction and stiffening of the vascular wall. Factors such as genetic predispositions, hypoxia and, mainly, pulmonary inflammation lead to modeling of the lung parenchyma, with heart failure and death being the most common outcomes of the untreated disease (Bao et al., 2023). PAH can present in variable forms, since the accumulation of smooth muscle cells in the pulmonary artery and inflammatory cell infiltrate can have their activity modeled according to the treatment performed (Humbert et al., 2019; Johnson et al., 2023). In addition, it is clear that pulmonary arterial hypertension (PAH) constitutes a complex condition, associated with the development of type 2 diabetes (Kim et al., 2015; Johnson et al., 2020), a condition notoriously related to inflammation and chronic stress.

For many patients who develop respiratory failure, mechanical ventilation becomes a recommended therapeutic resource. However, if its indication is inappropriate, in severe cases of the patient or in which the parameters are not adjusted correctly, inflammation may worsen. Thus, the use of steroid anti-inflammatory drugs, such as dexamethasone, may be recommended for the treatment of failure due to its action in inhibiting factors AP-1, NF-кB, reducing the production of cytokines IL-1, IL-2, IL-3, IL-5, IL-6, IL-8, IL-12, tumor necrosis factor α (TNF-α), interferon γ (IFN-y), leukocyte colony growth factors and pulmonary endothelial growth factors (Rhen and Cidlowski, 2005). One point to be raised is that dexamethasone does not show activity against alveolar-capillary barrier dysfunction in animal models of lung injury due to mechanical ventilation, in addition to the increase in the volume of extravascular pulmonary fluid in animal models of lung injury due to hyperoxia (Reis et al., 2016). This fact shows that other mechanisms, in addition to the classic inflammation mediated by cytokines and leukocytes, participate in the development of lung injury.

Pulmonary disorders, exemplified by COPD, idiopathic pulmonary fibrosis (IPF) and asthma, may be susceptible to the exacerbated influence of AH, since the systemic inflammatory response propagates in the lung tissues, exacerbating the inflammation intrinsic to these diseases. In this context, the interaction between AH and pulmonary pathology is synergistic, potentiating the clinical manifestations of progression of the underlying lung disease (Imaizumi et al., 2014). PAH is an example of this interaction, exhibiting mechanisms of vascular remodeling, inflammation and endothelial dysfunction, imposing additional overload on the heart (Arkani et al., 2023).

Worldwide, hypertension is recognized as a risk factor for the development of cardiovascular and respiratory complications, and epidemiological studies have indicated a high prevalence of this condition among hospitalized patients. Thus, this relationship between hypertension and inflammatory lung disease is associated with the severity of symptoms in patients with COVID-19 in hypertensive patients. In a study carried out in 2020 in Italy with 1,043 hospitalized patients, it was shown that approximately 509 (49%) of them had hypertension. It stood out as the most common comorbidity among the participants, followed by cardiovascular disease (21%) and hypercholesterolemia (18%). In addition, the prevalence of hypertension was higher among patients who died in the intensive care unit (ICU) (63%, 195 of 309), representing the importance of the condition for their worse prognosis (Grasselli et al., 2020).

In the same year, another study characterizing 5,700 patients hospitalized with COVID-19 in the United States showed that 56.6% (3,026) had hypertension, which was also the most common comorbidity, followed by obesity (1,737, 41.7%) and diabetes (1,808, 33.8%) of cases (Richardson et al., 2020). It also suggested that the pre-existence of hypertension is an important worsening factor, since due to the degree of complication, there was a lower probability of using invasive mechanical ventilation as therapy for hypertensive patients. Also in 2020, the Chinese Center for Disease Control published that of 44,672 patients hospitalized with COVID-19, 13% were hypertensive, with this comorbidity being present in 40% of those who died (Guan et al., 2020). The correlation between worsening COVID-19 symptoms and hypertension involves complex mechanisms, with three hypotheses discussing their interplay. The first hypothesis relates to the ability of the cytokine storm during COVID-19 to exacerbate the systemic inflammatory condition of hypertension already present in the patient, with hypertension itself being a precursor to such a previous storm (Rodgers and Gibbons, 2020; Barroso et al., 2021). The second hypothesis would be related to the fact that hypertension is frequently associated with DM and CVD, which are also considered risk factors for severe complications of COVID-19 (Grasselli et al., 2020; Sardinha et al., 2021).

**FIGURE 3:**
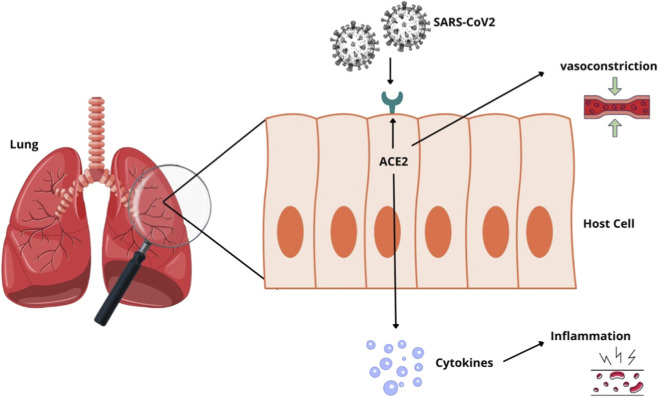
Correlation mechanisms between SARS-CoV-2 infection and arterial hypertension. The image demonstrates the entry of the virus into the host cell mediated by the angiotensin-converting enzyme 2 (ACE-2). After the onset of infection, there is the release of several pro-inflammatory cytokines, known as a cytokine storm, so that ACE-2 is also implicated in vasoconstriction and increased blood pressure.

Finally, a third hypothesis concerns the involvement of angiotensin-converting enzyme 2 (ACE2) in hypertension and COVID-19. The acute respiratory syndrome virus 2 (SARS-CoV-2) is known to belong to the coronavirus family, which includes viruses responsible for the common cold and mild pneumonia, severe acute respiratory syndrome (SARS) virus, and severe Middle East respiratory syndrome (MERS) virus. As shown in Figure 3, SARS-CoV-2 mainly injures the respiratory system, but is not restricted to it, due to its interaction with ACE2 for entry into host cells (Zhu et al., 2020; Laue et al., 2021; Bardelcíková et al., 2022). Hypertensive patients have altered expression of ACE2, which may be an explanation for the large number of hypertensive patients with complications from COVID-19 (Kulkarni et al., 2020; Bosso et al., 2020).

**FIGURE 4:**
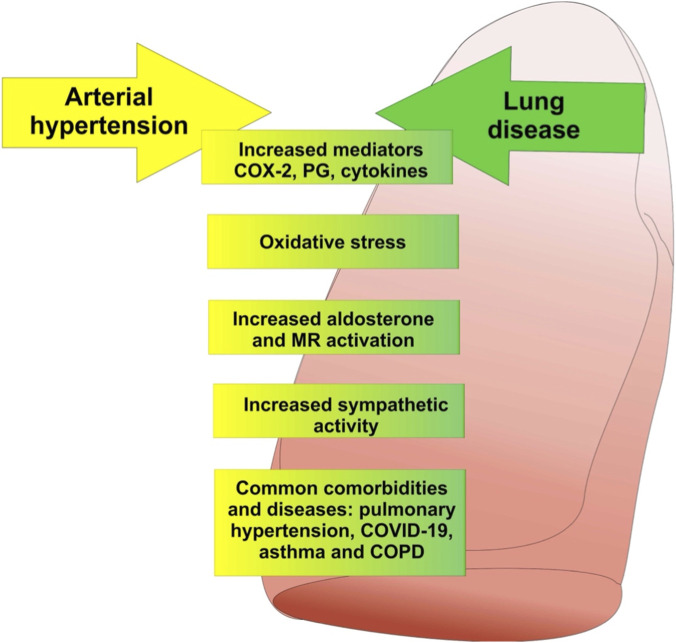
Interconnections between Arterial Hypertension and Lung Diseases. This image illustrates the complex interaction between arterial hypertension and lung diseases, highlighting the biological mechanisms that connect these conditions. The figure describes how hypertension can exacerbate lung problems through the increase of mediators such as COX-2, prostaglandins and cytokines, which induce oxidative and intense stress. The elevated presence of aldosterone and the activation of mineral receptors (MR) decrease to aggravate these processes. In addition, increased sympathetic activity can intensify the inflammatory and vascular response in these conditions. The image also highlights the common comorbidities between hypertension and lung diseases, including COVID-19, asthma and COPD, highlighting the interdependence between hypertension control and the management of chronic respiratory diseases.

In general, a thorough understanding of the molecular and cellular mechanisms involved in lung inflammation is essential for the development of targeted therapeutic strategies, aiming to selectively modulate the immune response and minimize the damage associated with lung injury. Thus, Figure 4 presents the different correlations between HA and inflammatory lung disease, which will be discussed below. In both conditions, there are common risk factors and a correlation between the severity of respiratory pathologies and increased blood pressure. There is also an association due to an increase in inflammatory mediators, expression of cyclooxygenase 2 (COX-2), as well as prostaglandins (PG) and cytokines, with free radicals being important in the induction of oxidative stress. There is also an increase in the function of aldosterone at its mineralocorticoid receptor (MR), sympathetic activity.

The human body naturally produces ROS and has antioxidant defense systems to neutralize their harmful effects. When imbalance occurs due to excessive, prolonged, or inappropriate production, the antioxidant and repair systems are overwhelmed, resulting in oxidative stress. The main ROS include superoxide radicals (O_2_^−^), hydroxyl radicals (OH^−^), and hydrogen peroxide (H_2_O_2_), causing damage to cell membranes, DNA, and biomolecules (Sies and Jones, 2020). Although not all the pathophysiological effects of ROS are known, they participate in the development of chronic inflammatory lung diseases (Rogers and Cismowski, 2018) and are involved in the pathophysiology of most viral respiratory infections, including influenza (Ng et al., 2014), respiratory syncytial virus (Martínez et al., 2016), rhinovirus (Kaul et al., 2000), and SARS-CoV-2 (Chernyak et al., 2020).

In COVID-19, infection increases ROS production through two main mechanisms: first, by activating NADPH oxidase (NOX) enzymes due to reduced bioavailability of ACE2 utilized by the virus (Laue et al., 2021); second, by mitochondrial impairment that induces elevated production of mitochondrial reactive oxygen species (mtROS) (Saleh et al., 2020). Downregulation of ACE2 activates the angiotensin II/AT1R/NADPH oxidase axis, producing O_2_^−^ and forming ONOO^−^, which inactivates the electron transport chain, further increasing mtROS production and depleting ATP levels (Doughan et al., 2008; Korge et al., 2016; Srivastava et al., 2019).

Another important pathway implicated in the relationship between pulmonary inflammation and hypertension involves the action of mineralocorticoids and their receptors. Discovered over 50 years ago, aldosterone is an important mineralocorticoid of the retinoid/thyroid/steroid ligand-dependent transcription factor superfamily, with action on sodium hemostasis in the body and, consequently, maintenance of blood pressure. Its action derives from the mineralocorticoid receptor (MR) expressed in the epithelium of the distal colon and nephron (Fuller et al., 2000). It is known that dysregulated expression of sodium channels can lead to dehydration of the airways of surfactant fluid, mucus and decreased local clearance, contributing to the progression of COPD and cystic fibrosis (Myerburg et al., 2006).

Another important point concerns the high aldosterone concentration in patients with PAH, which is directly proportional to the severity of clinical symptoms. Studies show that MR contributes to pulmonary vascular inflammatory infiltrate in PAH. This activity derives from MR activation and induction of increased TNF-α, intracellular adhesion molecule 1 (ICAM-1), connective tissue growth factor (CTGF), metalloproteinase 2 (MMP2) and 9 (MMP9) and pulmonary vascular remodeling. In this sense, blockade of the MR-aldosterone pathway in *in vivo* and *in vitro experiments* demonstrates that there is a reduction in pulmonary vascular remodeling and a decrease in pulmonary arterial pressure (Blanner et al., 2007; Maron et al., 2012; Tu et al., 2022).

Furthermore, it is known that pulmonary artery endothelial cells express MR and that it is physiologically associated with the induction of endothelial nitric oxide synthase (eNOS) activity. Once activated, eNOS induces the production of nitric oxide (NO), a molecule that, in addition to having local hypotensive activity, regulates inflammation and thrombosis. During PAH, there is a lack of control of the NO production pathway, resulting from high aldosterone concentrations and ROS levels (Maron et al., 2012; Mamazhakypov and Lother, 2023). The importance of NO is evidenced by the fact that the United States Food and Drug Administration uses inhaled NO for the treatment of neonatal PAH (Aguilar et al., 2019).

Another hypothesis for the association between hypertension and pulmonary inflammation is the autonomic nervous system, due to its ability to modulate the vascular response. During hypertension (Esler, 2000) and PAH (Velez-Roa et al., 2004), there is an increase in sympathetic activity, highlighted by the increase in body vascular activity and increased pressure. Along with this observation, it is also known that activation of the sympathetic system is correlated with activation of the immune system (Kenney and Ganta, 2014), especially interstitial macrophages residing near the sympathetic nerves in the bronchovascular bundle (Ural et al., 2020).

Chronic inflammation and oxidative stress are the central pathogenic mediators that interconnect and exacerbate diabetes mellitus, hypertension, and lung disease. In diabetes, insulin resistance and endothelial dysfunction are potentiated by chronic hyperglycemia, which amplifies systemic inflammation. This inflammatory increase is driven by the production of proinflammatory cytokines (IL-6, TNFα) and directly compromises both lung function and the prognosis of hypertensive patients, demonstrating a complex systemic interdependence (Zhuravlyova and Kulikova, 2019; Khateeb et al., 2019; Aronson and Podolanczuk, 2021).

Functional and structural alterations of the bronchovascular bundle in patients with arterial hypertension (AH) or pulmonary hypertension (PI) are primarily driven by inflammatory processes and dysregulation of vascular homeostasis. These mechanisms are severely exacerbated by chronic hyperglycemia in diabetes mellitus, which overlaps with preexisting damage, amplifying oxidative stress and activating inflammatory pathways in various tissues (Zhu et al., 2020; Laue et al., 2021). Diabetes, through sustained glucose levels, acts as an additional factor in tissue damage, intensifying the effects of AH and PI. This impact is evident in cellular signaling, where excess glucose triggers inflammatory cascades, such as activation of the NFκB pathway, a topic that requires further analysis of the impact of diabetes on vascular and pulmonary tissues (Kulkarni et al., 2020; Bosso et al., 2020).

Persistent hyperglycemia drives vascular and tissue dysfunction through the activation of inflammatory and metabolic pathways, with the NFκB pathway being central to this process. Excess glucose may contribute to the activation of PKC and increased oxidative stress, generating ROS that directly activate NFκB. This transcription factor stimulates the intense production of pro-inflammatory cytokines (TNFα, IL-6, IL-1β), intensifying inflammation (Aghasafari et al., 2019). Chronic activation is associated with, or suggests a potential mechanism for, increased endothelial permeability, impaired NO-mediated vasodilation, and vascular remodeling, and promotes fibrosis and thickening of the bronchovascular bundle in the lungs (Aguilar et al., 2019). The correlation between elevated glucose and inflammatory markers suggests a higher risk of cardiovascular and pulmonary complications in diabetics, and strict glycemic control can mitigate the damage by reducing NFκB activation. It is noteworthy that the coexistence of arterial hypertension (AH) and diabetes significantly worsens vascular remodeling, highlighting the need for early interventions (Bao et al., 2023; Karabaeva et al., 2024; Jia et al., 2024; Chen et al., 2018).

Chronic hyperglycemia compromises lung health, intensifying inflammation and tissue remodeling by causing a significant increase in oxidative stress. This stress results from the mitochondrial overproduction of Reactive Oxygen Species (ROS), triggered by exacerbated glycemic metabolism (Christiansen et al., 2016). ROS are pathogenic through a dual pathway: they damage cellular structures and impair endothelial function by reducing the bioavailability of nitric oxide (NO). This reduction simultaneously contributes to hypertension and the worsening of lung diseases. Clinical studies confirm this direct link between inadequate glycemic control and increased inflammation and worsening respiratory function (Aronson and Podolanczuk, 2021).

Reactive oxygen species (ROS) directly impact nitric oxide (NO) signaling, a crucial molecule for endothelial function by promoting vasodilation and inhibiting cell adhesion. In the presence of excess ROS, NO is rapidly inactivated and converted to peroxynitrite, a highly reactive species that causes structural damage by degrading proteins, lipids, and DNA in vascular tissue. This reaction reduces NO bioavailability, and dysfunction of the eNOS (endothelial nitric oxide synthase) enzyme, often induced by oxidative stress, further exacerbates this deficiency. This vicious cycle sustains endothelial dysfunction and exacerbates hypertension (Doughan et al., 2008).

In the pulmonary context, the deleterious effects of Reactive Oxygen Species (ROS) transcend the vascular system, directly affecting the architecture and function of respiratory tissue. This inflammatory response, intensified by oxidative stress, suggests a potential interaction that exacerbated recruitment of immune cells, culminating in chronic inflammation and tissue remodeling. Direct consequences include the formation of fibrous tissue, thickening of the extracellular matrix, and impaired lung elasticity (Doughan et al., 2008).

Case studies confirm that inadequate glycemic control in diabetics suggests a potential interaction involving the elevated levels of inflammatory markers, reduced pulmonary diffusion capacity, and worsening of conditions such as pulmonary hypertension and chronic obstructive pulmonary disease. These clinical findings reinforce the direct link between chronic hyperglycemia, oxidative stress, and endothelial and pulmonary impairment, emphasizing the importance of rigorous glycemic management for prevention (Esler, 2000).

The polyol pathway, a compensatory metabolic pathway activated by chronic hyperglycemia in diabetes, significantly contributes to the progression and worsening of complications such as hypertension and lung disease. In this pathway, the enzyme aldose reductase converts excess glucose into sorbitol, consuming NADPH. Even though sorbitol is oxidized to fructose, the hyperflux of this mechanism induces osmotic imbalances and increases oxidative stress, generating critical cellular imbalances (Ferguson et al., 2014; Zheng et al., 2025). These processes exacerbate endothelial dysfunction and insulin resistance, creating an environment conducive to the development of microvascular and macrovascular complications that severely impact lung function and hypertension (Rosano et al., 2021).

In parallel, fructose production enhances the formation of advanced glycation end products (AGEs) and increases the activity of inflammatory pathways. These AGEs interact with their receptors (RAGEs) on endothelial cells, activating inflammatory cascades and contributing to additional oxidative stress. In the vascular endothelium, these changes exacerbate endothelial dysfunction by further reducing nitric oxide bioavailability and promoting the adhesion of pro-inflammatory molecules, such as ICAM-1 and VCAM-1. This inflammatory and pro-oxidative environment is a key factor in the progression of microvascular diseases, such as diabetic nephropathy and retinopathy, and macrovascular complications, including atherosclerosis and hypertension (Myerburg et al., 2006; Rigalleau et al., 2021; Poznyak et al., 2020).

In the pulmonary context, the impact of the polyol pathway is equally significant. Chronic hyperglycemia promotes inflammation and tissue remodeling, processes amplified by the increase in osmotic and oxidative stress associated with the activation of the polyol pathway. The accumulation of sorbitol and the consequent osmotic imbalance compromise the endothelial and epithelial cells of the lungs, favoring fibrosis, thickening of the pulmonary vascular walls and the development of pulmonary hypertension. In addition, increased fructose production can intensify local and systemic inflammation, aggravating respiratory and cardiovascular diseases concomitantly (Narumiya et al., 1999; Li et al., 2021).

These mechanisms demonstrate that chronic activation of the polyol pathway is not only an adaptive response to excess glucose, but a pathological factor that aggravates the progression of micro- and macrovascular complications in diabetes. Detailed understanding of these molecular processes highlights the importance of strict glycemic control as a central strategy to minimize the deleterious effects associated with this metabolic pathway and prevent the progression of systemic chronic diseases (Ng et al., 2014).

Diabetes treatments have a direct and indirect influence on hypertension and lung disease, while therapies targeting these comorbidities also affect diabetes. For example, SGLT2 inhibitors have shown benefits in glycemic control and blood pressure reduction, in addition to anti-inflammatory effects that may protect lung function. On the other hand, medications such as beta-blockers, widely used for hypertension, can negatively influence glycemic control, requiring personalized therapeutic adjustments. This interrelation reinforces the need for an integrated approach in the clinical management of patients with these conditions (Bozkurt et al., 2021).

The clinical implications of these interactions highlight the importance of management strategies that recognize the interconnection between diabetes, hypertension, and lung disease. Holistic therapeutic interventions should prioritize not only glycemic and blood pressure control, but also the modulation of systemic inflammation and oxidative stress. Integrated rehabilitation programs and the use of biomarkers to monitor these interactions could optimize therapeutic outcomes, while novel therapeutic targets, such as modulation of the polyol pathway, could provide additional solutions to minimize associated complications (Bozkurt et al., 2021; Arkani et al., 2023).

However, it is clear that effective management of patients with diabetes and its comorbidities must be based on a deep understanding of the shared pathophysiological pathways, promoting a personalized and multidisciplinary approach. This integrated model not only improves the quality of life of patients but also reduces the risk of serious complications associated with the interactions between these conditions (Donath et al., 2019; Vaduganathan et al., 2022).

### 3.2 Pathophysiological connections between diabetes, arterial hypertension and pulmonary inflammation

The relationship between arterial hypertension (AH), pulmonary inflammation (PI) and diabetes mellitus is complex and multifaceted. Regarding the analysis on AH and PI, it is important to highlight how these conditions interact and intertwine, especially with regard to the role of chronic inflammation and oxidative stress as demonstrated in Supplementary Table S3. These two characteristics are common in both hypertension and pulmonary inflammation and are decisively crucial in the pathogenesis of diabetes mellitus (Vaduganathan et al., 2022).

Chronic inflammation is a central factor in the development of type 2 diabetes. Increased inflammatory cytokines, often associated with hypertension and lung disease, contribute to insulin resistance and beta-cell dysfunction. Additionally, the oxidative stress resulting from these processes worsens glycemic homeostasis, perpetuating the vicious cycle of diabetes (Vaduganathan et al., 2022).

Chronic hyperglycemia in diabetes triggers mitochondrial dysfunction and the overproduction of reactive oxygen species (ROS). This metabolic overload intensifies the activity of the electron transport chain, promoting electron escape (in complexes such as I and III) and excessive superoxide formation, which compromises mitochondrial integrity and damages essential biomolecules (Sies and Jones, 2020). The accumulation of this damage establishes a negative feedback loop, in which reduced respiratory efficiency exacerbates ROS generation. This subsequent dysfunction compromises the organelle’s dynamics and mitophagy, culminating in the release of pro-apoptotic mediators (such as cytochrome c) and the loss of cells critical for homeostasis, such as cardiomyocytes, endothelial cells, and pancreatic beta cells (Silva et al., 2023).

The systemic impact of mitochondrial dysfunction is amplified by its influence on cell signaling and inflammation. This inflammatory environment intensifies insulin resistance in peripheral tissues (muscle and liver), perpetuating hyperglycemia. In the lungs, ROS exacerbate the recruitment of inflammatory cells, alter vascular permeability, and promote tissue remodeling, contributing to conditions such as pulmonary hypertension and fibrosis (Snyder and Farber, 2019).

The connection between mitochondrial dysfunction and cardiovascular and respiratory pathologies is also evident. In the vascular endothelium, oxidative stress reduces the bioavailability of nitric oxide (NO), compromising vasodilation and promoting endothelial dysfunction, a key precursor of hypertension. In lung tissues, mitochondrial dysfunction alters the energy metabolism of epithelial and endothelial cells, compromising the barrier function and favoring fibrotic processes. Furthermore, exacerbated apoptosis in vascular and smooth muscle cells contributes to the structural remodeling observed in hypertensive and pulmonary diseases (Brazilian Society of Cardiology, 2017).

Finally, the perpetuation of the cycle of hyperglycemia, oxidative stress and inflammation reflects a complex interplay between metabolic and cellular factors, highlighting mitochondrial dysfunction as a central link in the progression of diabetes and its associated complications. A detailed understanding of these molecular mechanisms is essential for the development of targeted therapies that can restore mitochondrial function, mitigate ROS production and interrupt the inflammatory-metabolic cycle, slowing or preventing the progression of cardiovascular and pulmonary diseases associated with diabetes (Stoppelaar et al., 2014).

It is therefore essential to understand how diabetes not only impacts inflammatory and metabolic responses in patients with hypertension and lung disease, but also how these conditions can worsen glycemic control. The simultaneous presence of diabetes, hypertension, and lung inflammation can lead to more severe complications, highlighting the importance of an integrated clinical management that addresses these conditions holistically (Aghasafari et al., 2019).

### 3.3 Pathophysiological mechanisms of diabetes

A detailed analysis of the intersections of diabetes in the modulation of inflammatory and metabolic responses associated with arterial hypertension and lung diseases, highlighting the markers involved, their interactions and the underlying pathophysiological mechanisms is shown in Supplementary Table S4. These markers do not act in isolation, but in an interconnected manner, exacerbating chronic conditions and enhancing the progression of cardiovascular and lung diseases.

Increased inflammatory cytokines (IL-6, TNFα, IL-8, MCP-1, IL-1β) are crucial for immune communication and exacerbate the pathogenesis of hypertension, pulmonary inflammation, and diabetes. These signaling molecules promote the recruitment of immune cells, and specifically, TNFα and IL-6 induce insulin resistance. Additionally, cytokines such as IL-1β, linked to the NLRP3 inflammasome, intensify chronic inflammation and lung and vascular tissue remodeling, driving the progression of these diseases (World Health Organization, 2021; Zhuravlyova and Kulikova, 2023).

Oxidative stress, defined as the overproduction of Reactive Oxygen Species (ROS), is a critical mechanism that destabilizes homeostasis. Under hyperglycemia and chronic inflammation, ROS damage biomolecules (DNA, lipids, proteins), compromising endothelial function and increasing vascular stiffness. In lung tissue, oxidative stress intensifies inflammation and promotes tissue remodeling, worsening pulmonary hypertension. This ongoing oxidative damage reduces cellular antioxidant capacity (e.g., decreased glutathione), perpetuating the cycle of systemic damage (Pouvreau et al., 2018; Rodgers and Gibbons, 2020).

Insulin resistance, amplified by inflammatory cytokines, impairs efficient glucose utilization, leading to blood glucose accumulation. This hyperglycemia may contribute to increased ROS production and activation of inflammatory pathways. Systemically, insulin resistance intensifies the progression of diabetes and hypertension and compromises respiratory capacity and glycemic control in metabolic tissues such as muscle and liver (Rogers and Cismowski, 2018; Mancusi et al., 2020; Lee, 2021; Jia and Sowers, 2021).

Endothelial dysfunction is a direct consequence of the interaction between oxidative stress and inflammation. Reduced nitric oxide (NO) bioavailability compromises vascular permeability and results in vasoconstriction, increasing blood pressure and cardiovascular risk. In the lungs, this dysfunction favors chronic inflammation, fibrosis, and pulmonary hypertension, worsening the condition in patients with diabetes and respiratory diseases (Saleh et al., 2020; Srivastava et al., 2019).

Finally, metabolic pathways, such as NF-kB and NLRP3, are at the center of inflammatory regulation and immune response. NF-kB activation stimulates the production of pro-inflammatory cytokines and amplifies the inflammatory response, while the NLRP3 inflammasome intensifies the release of IL-1β, perpetuating the inflammatory cycle (Wang et al., 2024). Alterations in insulin signaling pathways, such as inadequate phosphorylation of proteins in the PI3K/Akt cascades, directly impact glycemic control and energy metabolism, worsening diabetes management. Chronic activation of these metabolic pathways reinforces the connection between diabetes, hypertension, and lung diseases, highlighting the need for targeted therapeutic interventions to interrupt these pathological cycles and improve patient prognosis (Sardinha et al., 2021; Wang et al., 2021).

Chronic inflammation and oxidative stress play crucial roles in the development of diabetes mellitus, particularly when considered in the context of conditions such as arterial hypertension (AH) and pulmonary inflammation (PI). The interrelationship between these conditions is complex, involving mechanisms that include insulin resistance and pancreatic beta cell dysfunction. Exacerbated oxidative stress, resulting from chronic inflammation and accumulation of reactive oxygen species, can induce metabolic alterations that link diabetes to hypertension and pulmonary diseases, generating a vicious cycle that worsens the clinical status of patients (Aghasafari et al., 2019; Nizamitdinovich and Toshtemirovna, 2021).

Insulin resistance, often associated with diabetes, can be precipitated by inflammatory mediators, such as proinflammatory cytokines, which interfere with insulin signaling and compromise the body’s ability to utilize glucose effectively. In addition, beta cell dysfunction resulting from oxidative stress and chronic inflammation can reduce insulin secretion, exacerbating hyperglycemia and contributing to the progression of comorbidities. Activation of oxidative stress-related signaling pathways, such as the polyol pathway, also plays a significant role, as hyperglycemia can increase the conversion of glucose to sorbitol using the enzyme aldose reductase, leading to microvascular complications and contributing to endothelial dysfunction (Sardinha et al., 2021).

The complexity of the interrelationships between hypertension, diabetes, and pulmonary inflammation lies in the fact that these pathologies share and mutually exacerbate common pathophysiological and molecular mechanisms. This mutual progression is sustained by a pathological cycle of chronic inflammation, oxidative stress, and metabolic dysfunction (Sardinha et al., 2021; Ning et al., 2021).

Metabolic signaling pathways, notably NFκB and the polyol pathway, are central in the context of metabolic stress and inflammation, contributing significantly to the progression of hypertension and pulmonary inflammation. The NFκB pathway is activated by inflammatory cytokines (IL-6, TNFα) and ROS, translocating to the nucleus to regulate the expression of pro-inflammatory genes (IL-1β, MCP-1). This mechanism drives vasoconstriction, vascular remodeling, and pulmonary inflammatory recruitment (Saleh et al., 2020). Simultaneously, the hyperflux of the polyol pathway under hyperglycemia consumes NADPH (reducing antioxidant protection via glutathione) and generates osmotic stress. The loss of antioxidant capacity intensifies oxidative stress and endothelial dysfunction, and the resulting AGE formation interacts with RAGEs to feed back into NFκB activation, amplifying the inflammatory cycle and insulin resistance (Rogers and Cismowski, 2018; Raguraman et al., 2021).

Oxidative stress, mediated by the excessive production of reactive oxygen species (ROS), is a central factor in endothelial dysfunction, a common feature of all three pathologies. ROS oxidize nitric oxide (NO), reducing its bioavailability and compromising vasodilation, resulting in persistent vasoconstriction and elevated blood pressure, in addition to affecting vascular regulation and oxygen transport in the lungs (Rogers and Cismowski, 2018). This dysfunction is amplified by systemic inflammation, where cytokines such as IL-6 and TNFα promote insulin resistance and increase ROS production, establishing a vicious cycle. TNFα and IL-6 aggravate endothelial dysfunction and hypertension, while intensifying lung tissue remodeling (Rodgers and Gibbons, 2020). Additionally, chronic hyperglycemia intensifies lung inflammation via the production of ROS and AGEs, and compromises the activity of alveolar macrophages, key defense cells, which exacerbates chronic respiratory diseases and worsens gas exchange (Bao et al., 2023).

The integration of the pathophysiological mechanisms underlying arterial hypertension, diabetes, and pulmonary inflammation is associated with these conditions are deeply interconnected. A detailed understanding of these interactions is crucial for developing therapeutic strategies aimed at interrupting the pathological cycles (Barberà, 2013). This interconnection is evident by the overlap of inflammatory markers (TNFα, IL-6, IL-1β) in the three pathologies, suggesting a common systemic inflammatory state. Chronic hyperglycemia and oxidative stress act as key mediators, amplifying vascular inflammation and endothelial dysfunction. Specifically, oxidative stress and glycation, along with activation of the polyol pathway, link pulmonary pathology to cardiovascular dysfunction, exacerbating insulin resistance and perpetuating the pathological cycle (Bao et al., 2023).

Diabetes induces endothelial dysfunction, which aggravates hypertension and lung disease by compromising vasodilation and promoting a prothrombotic environment, increasing the risk of cardiovascular events (Aguilar et al., 2019; Rodgers and Gibbons, 2020). The interactions between these pathologies (diabetes, hypertension, and pulmonary inflammation) complicate clinical management, increase the risk of serious complications (such as heart failure and COPD), and require early treatment (Aguilar et al., 2019; Rogers and Cismowski, 2018; Tarar et al., 2024). However, diabetes treatment presents a therapeutic intersection: medications such as metformin demonstrate potential to positively impact inflammatory processes, offering benefits in the concomitant management of hypertension and pulmonary inflammation (Aguilar et al., 2019; Rodgers and Gibbons, 2020; Ruscica et al., 2020).

Population aging is a global trend that projects a significant increase in the number of long-lived elderly (Santos et al., 2024; Hambleton et al., 2023). Although positive, this scenario poses a challenge to public health, as aging implies a progressive decline in physiological efficiency and regenerative capacity (Pivetta and Zorzetto, 2017; Macena et al., 2018; Cochar-Soares et al., 2021). Molecularly, the process is linked to the accumulation of damage, such as oxidative stress, chronic low-grade inflammation (inflammaging), and cellular senescence, which compromise homeostasis and increase susceptibility to chronic noncommunicable diseases (NCDs) (Teixeira and Guariento, 2010; Pivetta and Zorzetto, 2017; Tersariol, 2021; d’Avila et al., 2020; Brunelli et al., 2024; Duft et al., 2024). Obesity, for example, accelerates immunosenescence (Bowker et al., 2020; McNeill et al., 2021; Brasil, 2024; Bekkering et al., 2024). Therefore, a deeper understanding of these mechanisms is crucial for developing strategies that ensure healthy and high-quality longevity.

## 4 Conclusion

This study characterized the complex immunological, molecular and pathophysiological interactions that interconnect arterial hypertension (AH), pulmonary inflammation (PI) and diabetes mellitus (DM), evidencing the existence of an intricate network of shared pathways that mutually exacerbate these chronic conditions. It was demonstrated that chronic inflammation and oxidative stress act as central axes in this interaction, creating a vicious cycle that perpetuates vascular dysfunction and tissue remodeling.

Specifically, hypertension has been shown to aggravate PI by inducing systemic inflammatory responses, activating NF-κB, and increasing proinflammatory cytokines such as TNF-α, compromising pulmonary vascular integrity. Concomitantly, chronic hyperglycemia in DM intensifies these processes by inducing oxidative stress, endothelial dysfunction, and reducing nitric oxide bioavailability, effects potentiated by activation of the renin-angiotensin-aldosterone system and the mineralocorticoid receptor. PI, in turn, mediated by macrophages and neutrophils, contributes to pulmonary vascular remodeling and impaired gas exchange, aggravating hypertension and DM.

The identification of central inflammatory mediators, such as NFκB and TNFα, highlights the urgency of integrated therapeutic strategies. Interventions such as SGLT2 inhibitors represent a promising avenue for modulating inflammation, oxidative stress, and vascular remodeling, mitigating the interdependent impacts of hypertension, PI, and DM.
