# Supplementary material for: Molecular and pathophysiological intersections between arterial hypertension, pulmonary inflammation and diabetes mellitus
Source: Front Pharmacol. 2026 Apr 1;16:1734861. doi: 10.3389/fphar.2025.1734861 (PMC13079043; doi:10.3389/fphar.2025.1734861)
Supplement: Supplementary file 1 [file Supplementaryfile1.pdf]

Supplementary Material

MOLECULAR AND PATHOPHYSIOLOGICAL INTERSECTIONS BETWEEN ARTERIAL HYPERTENSION, PULMONARY INFLAMMATION AND DIABETES MELLITUS

1 Graphical Abstract

The graphical abstract presented schematically illustrates the main molecular and pathophysiological interconnections between arterial hypertension, pulmonary inflammation, and diabetes mellitus identified in this scoping review, providing an integrated visual representation of the study's main findings.

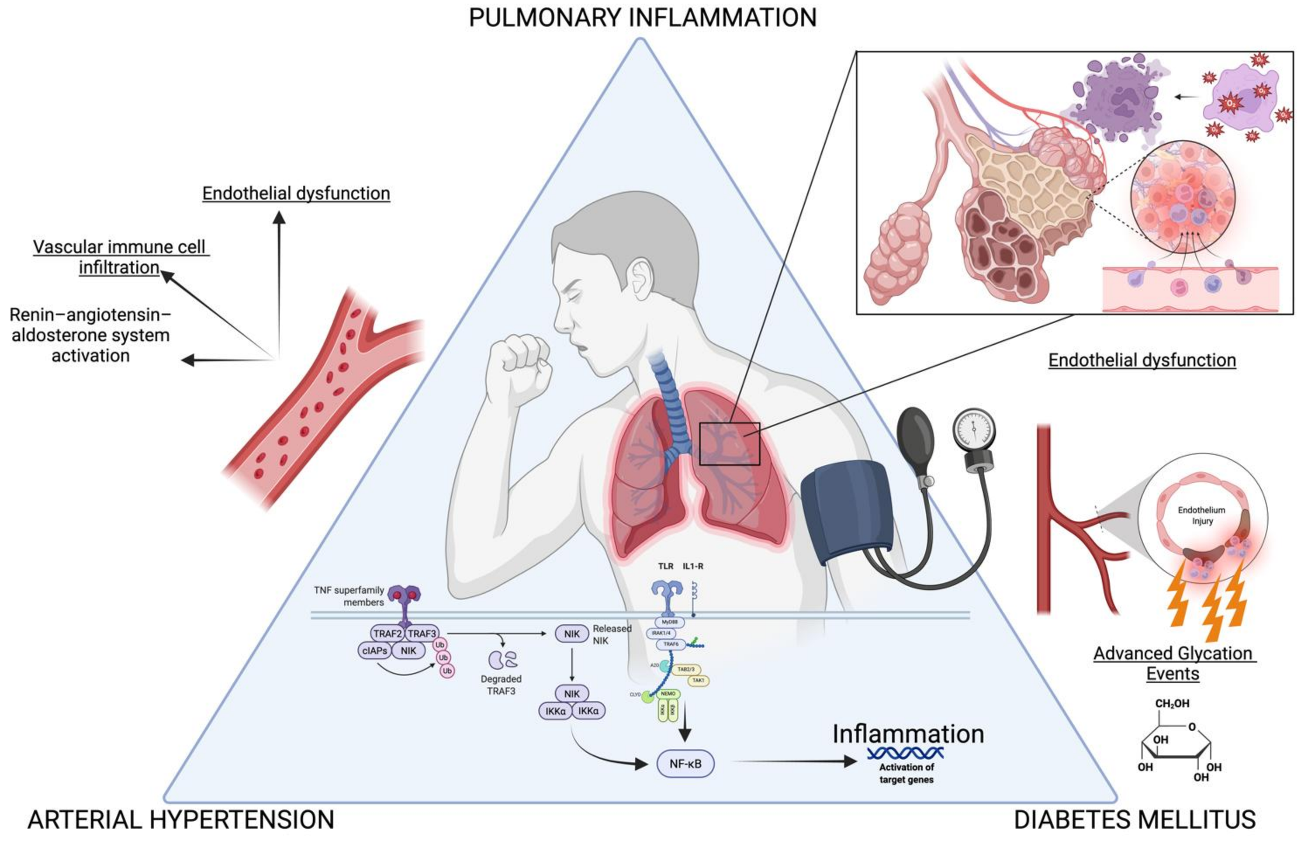

Common inflammatory and oxidative pathways link hypertension, diabetes and pulmonary inflammation activation.

Graphical Abstract – Molecular intersections linking arterial hypertension, pulmonary inflammation, and diabetes mellitus.

Chronic inflammation and oxidative stress act as shared molecular axes connecting arterial hypertension, pulmonary inflammation, and diabetes mellitus. Activation of the renin–angiotensin–

aldosterone system (RAAS), endothelial dysfunction, and immune cell infiltration promote vascular inflammation and remodeling. Concurrently, hyperglycemia induces advanced glycation events and oxidative stress, reducing nitric oxide bioavailability and amplifying NF-κB–mediated inflammatory signaling. Together, these mechanisms establish a systemic pro-inflammatory state that perpetuates vascular injury and pulmonary dysfunction. Source: Created by the authors using BioRender.com.
