# Supplementary material for: Molecular and pathophysiological intersections between arterial hypertension, pulmonary inflammation and diabetes mellitus
Source: Front Pharmacol. 2026 Apr 1;16:1734861. doi: 10.3389/fphar.2025.1734861 (PMC13079043; doi:10.3389/fphar.2025.1734861)
Supplement: Supplementary file 2 [file Supplementaryfile2.pdf]

Supplementary Material

MOLECULAR AND PATHOPHYSIOLOGICAL INTERSECTIONS BETWEEN ARTERIAL HYPERTENSION, PULMONARY INFLAMMATION AND DIABETES MELLITUS

1 Supplementary Tables

This supplementary material contains detailed information that supports the scoping review presented in the main article. The tables below provide complete search strategies for each database and comprehensive characteristics of all included studies, complementing the methodology and results discussed in the manuscript.

Table 1 – Research strategies for articles in databases and their search descriptors.

Databases	Search Descriptors
**MEDLINE**	((((((Hypertension[MeSH Terms]) OR (pulmonary inflammation[MeSH Terms])) OR (lung disease[MeSH Terms])) OR (Chronic respiratory diseases[MeSH Terms])) OR (diabetes and hypertension[MeSH Terms])) OR (Diabetes and pulmonary inflammation[MeSH Terms])) OR (Diabetes and lung disease[MeSH Terms])
**Academic Google**	"Hypertension" OR "pulmonary inflammation" OR "lung disease" OR "Pulmonary hypertension" OR "Chronic respiratory diseases" OR "diabetes and hypertension" OR "Diabetes and pulmonary

	inflammation" OR "Diabetes and lung disease"
**Web of Science**	TS=("Hypertension" OR "pulmonary inflammation" OR "lung disease" OR "Pulmonary hypertension" OR "Chronic respiratory diseases" OR "diabetes and hypertension" OR "Diabetes and pulmonary inflammation" OR "Diabetes and lung disease")
**EMBASE**	('hypertension'/exp OR 'pulmonary inflammation'/exp OR 'lung disease'/exp OR 'pulmonary hypertension'/exp OR 'chronic respiratory diseases'/exp OR 'diabetes and hypertension'/exp OR 'diabetes and pulmonary inflammation'/exp OR 'diabetes and lung disease'/exp)
**ScienceDirect**	TITLE-ABS-KEY("hypertension" OR "pulmonary inflammation" OR "lung disease" OR "Pulmonary hypertension" OR "Chronic respiratory diseases" OR "diabetes and hypertension" OR "Diabetes and pulmonary inflammation" OR "Diabetes and lung disease")

Caption: This table presents the specific search strategies employed across different electronic databases (MEDLINE, Academic Google, and Web of Science) during the scoping review. The search descriptors combine Medical Subject Headings (MeSH) terms and keywords related to hypertension, pulmonary inflammation, lung diseases, diabetes, and their interconnections. Boolean operators (OR, AND) were used to create comprehensive search strings tailored to each database's indexing system to ensure maximum retrieval of relevant studies addressing the relationship between these pathological conditions.

Table 2 – Scientific Studies on Hypertension and Pulmonary Inflammation: Molecular and Clinical Approaches.

Nº	Title	Author, Year	Journal	Description/Key Information
1	Factors associated with systemic hypertension in asthma	Ferguson *et al*., 2014	Lung	The study found a significant association between hypertension and reduced lung function (measured by FEV1%), obstructive sleep apnea (OSA) and the use of inhaled corticosteroids. Inflammatory markers such as TNF-α, IL-6, CRP (C-reactive protein) and other indicators of systemic inflammation were found to be risk factors for hypertension in asthmatic patients. The study suggests that adequate control of airway inflammation and early detection of apnea may reduce the risk of hypertension in this population.
2	Lung Disease and Hypertension	Imaizumi, Eguchi e Kario, 2014	Pulse (Basel)	The article addresses how lung inflammation is associated with increased sympathetic activity, which may contribute to the development and worsening of hypertension. This link is essential to understanding the elevated cardiovascular risks in patients with lung disease. In addition, the text highlights inflammatory markers such as TNF-α and IL-6, which are elevated in

				these patients, indicating an inflammatory response that may act as a link between hypertension and lung conditions.
3	Autonomic nervous system and immune system interactions	Kenney e Ganta, 2014	Comprehensive Physiology	The study highlights the critical role of neurotransmitters and immune receptors in inflammatory diseases, showing how immune cells, such as macrophages and lymphocytes, have adrenergic and nicotinic receptors. These receptors, when activated by neurotransmitters from sympathetic and parasympathetic nerve endings, trigger immunomodulatory actions. Inflammatory markers, such as IL-1β, IL-6, TNF-α and IFN-γ, are essential in mediating these responses and can intensify chronic conditions such as hypertension and pulmonary inflammation. The article highlights that the interaction between the nervous system and these inflammatory markers can both aggravate and be aggravated by such conditions.
4	Prevalence and impact of diabetes, hypertension, and cardiovascular diseases in chronic obstructive pulmonary diseases:	Mahishale *et al.*, 2015	International Journal of Translational Medicine	Research shows that high blood pressure (HBP) and lung inflammation are interconnected, with inflammation being a key factor in worsening HBP in COPD patients. Immune cells such as macrophages

	A hospital-based cross-section study			and lymphocytes respond significantly in these patients, exhibiting high levels of inflammatory markers such as TNF-α, IL-6, and other factors that exacerbate both lung inflammation and HBP.
5	Reactive oxygen species production in cardiac mitochondria after complex I inhibition: Modulation by substrate-dependent regulation of the NADH/NAD(+) ratio	Korge, Calmettes e Weiss, 2016	Free radical biology & medicine	Using alamethicin-permeabilized cardiac mitochondria, the authors show that ROS production is highly dependent on the substrates present, specifically malate and glutamate, which directly affect NADH generation through the coordinated activities of the enzymes malate dehydrogenase (MDH) and aspartate aminotransferase (AST). The research highlights the complex interplay between NADH generation and ROS production, underlining the importance of metabolic processes in regulating mitochondrial function and oxidative stress.
6	Hypertension and Asthma: A Comorbid Relationship	Christiansen *et al.,* 2016	The Journal of Allergy and Clinical Immunology	Patients with asthma and hypertension have greater severity of asthma symptoms, evidenced by increased use of beta-agonists, emergency room visits, and hospitalizations. The link between hypertension and asthma appears to be mediated by smooth muscle and endothelial cells, leading to vascular remodeling and endothelial dysfunction. This scenario highlights

				hypertension as a worsening factor for morbidity in diseases, requiring integrated management strategies for both conditions. The importance of inflammatory markers such as IL-6 and TNF-α, common in both disorders, highlights the interconnected complexity of these diseases and the need for coordinated treatment.
7	Oxidative Stress in the Lung - The Essential Paradox	Rogers e Cismowski, 2018	Current Oppnion in Toxicology	The article highlights the relationship between arterial hypertension and pulmonary inflammation, where changes in oxygen tension can lead to increased ROS production, aggravating pulmonary inflammation and promoting vascular remodeling. Immune cells such as macrophages and neutrophils play a critical role in modulating these responses, through the release of inflammatory markers such as IL-6, TNF-α, and nitric oxide, which are involved in both the inflammatory response and the processes of lung tissue damage and vascular remodeling.
8	Hypertension	Oparil et al., 2018	Nature Reviews Disease Primers	This article details the interaction between arterial hypertension and pulmonary inflammation, highlighting the role of immune cells and inflammatory markers such

				as NO, IL-17A, IFN-γ, IL-6, TNF-α, TGF-β, IL-1β, and NFKB. These markers are essential to understand the pathophysiology of hypertension and its impact on pulmonary inflammation, indicating that hypertension can exacerbate inflammatory conditions, contributing to vascular remodeling and damage to target organs such as the heart and kidneys.
9	Pathology and pathobiology of pulmonary hypertension: state of the art and research perspectives	Humbert *et al.*, 2019	European Respiratory Journal	This article reviews recent advances in understanding the pathology and cellular mechanisms that affect pulmonary vascular remodeling associated with various forms of pulmonary hypertension (PH). The influence of immune cells and inflammatory markers such as IL-1β, IL-6, TNF-α, and vascular remodeling observed in pathological states are highlighted. These inflammatory markers, together with vascular remodeling and vessel loss, are associated with increased pulmonary arterial pressure and pulmonary vascular resistance, which leads to progressive right heart failure.
10	Postnatal melatonin treatment modulates the expression of prostanoid agents in the lungs of newborn sheep with	Aguilar *et al.*, 2019	Chilean medical journal	This study explores the effects of melatonin on the expression of prostanoid pathways in the lungs of neonatal lambs under hypobaric conditions,

	pulmonary hypertension.			which induce neonatal pulmonary arterial hypertension (NPAH). Research highlights that melatonin treatment increases the expression of prostacyclin syntaxin (vasodilator) and reduces the expression of cyclooxygenase-2, markers that have implications for lung inflammation and hypertension. Immune cells, such as macrophages, may be affected by these immunological markers, influencing inflammation and vascular remodeling associated with arterial hypertension.
11	Human lung tissue resident memory T cells in health and disease.	Snyder e Farber, 2019	Current Opinion in Immunology	This article reviews the role of tissue-resident memory (TRM) T cells in the human lung, highlighting their importance in pulmonary immunity and pathology. TRM are predominantly found in the lungs and play roles in both the protective response against pathogens and in maintaining tissue homeostasis. Recent studies have identified that these cells expressed markers such as CD69 and CD103 and possessed a transcriptional profile that supported their residence and function in the tissue. Pulmonary TRM are implicated in both antitumor immune responses and chronic inflammatory pathologies, such as asthma. Associated inflammatory markers

				include IL-17A, IFN-γ, TNF-α, among others, decreasing their responsiveness and potentiating inflammatory responses in the context of arterial hypertension and pulmonary inflammation.
12	COVID-19 and hypertension	Kulkarni, Jennere Wilkinson, 2020	Journal of the Renin-Angiotensin-Aldosterone System	This article explores how the renin-angiotensin-aldosterone system (RAAS) and the interface between arterial hypertension and pulmonary inflammation may be exacerbated in the presence of SARS-CoV-2, with an emphasis on inflammatory markers such as angiotensin II, which is associated with inflammation and lung injury. It also details the controversial interaction between angiotensin-converting enzyme inhibitor (ACEI) drugs and angiotensin II receptor blockers (ARBs) in the context of COVID-19, revealing that the latter may alter the expression of ACE2, a receptor crucial for viral entry into cells, potentially affecting the severity of infection and related inflammatory responses.
13	Identification of a Nerve-Associated, Lung-Resident Interstitial Macrophage Subset With Distinct Localization and	Ural *et al*., 2020	Scientifc Imunology	It is noteworthy that alveolar macrophages, when interacting with apoptotic cells, require the presence of cytokines such as IL-4 or IL-13 to promote replacement parts and effective tissue remodeling.

	Immunoregulatory Properties			This interaction is crucial for the maintenance of adequate lung function, especially in contexts of tissue stress or damage. The article emphasizes the importance of understanding how these negative immune cells contribute to lung homeostasis and adaptive responses in lung disease settings and possible therapeutic implications.
14	Clinical Characteristics of Coronavirus Disease 2019 in China	Guan *et al.*, 2020	New England Journal of Medicine	The study analyzes the clinical characteristics of COVID-19 patients across China, focusing on immunological features and inflammatory markers. While the paper does not directly link high blood pressure to lung inflammation, it does detail the role of immune cells and inflammatory markers, such as lymphocytopenia (83.2% of patients) and elevated C-reactive protein levels. These markers suggest an intense and systemic immune response to the infection.
15	The Two Faces of ACE2: The Role of ACE2 Receptor and Its Polymorphisms in Hypertension and COVID-19	Bosso *et al.*, 2020	Molecular Therapy Methods & Clinical Development	This article explores the dual role of the ACE2 receptor, which is crucial both in modulating blood pressure and as a portal for the SARS-CoV-2 virus. It highlights the relevance of ACE2 gene polymorphisms, which may affect both susceptibility to hypertension and the severity of COVID-19.

				These findings suggest that genetic variations in ACE2 may be determinants of responses to hypertension treatment and of susceptibility and severity of SARS-CoV-2 infections.
16	Morphometry of SARS-CoV and SARS-CoV-2 particles in ultrathin plastic sections of infected Vero cell cultures	Laue *et al*., 2021	Scientific Reports	The article mentions that SARS-CoV-2 infection in Vero cell cultures can induce oxidative stress, resulting from increased production of reactive oxygen species (ROS). Oxidative stress, caused by ROS overload, can lead to significant damage to host cells, affecting biomolecules such as lipids, proteins, and DNA. Oxidative stress contributes to viral pathogenesis by compromising cellular integrity, promoting inflammation, and impacting the immune response, favoring viral replication and intensifying the inflammatory response.
17	Mineralocorticoid Receptor Antagonism by Finerenone Attenuates Established Pulmonary Hypertension in Rats	Tu *et al.,* 2022	Hypertensionaha	The study analyzed the role of the mineralocorticoid receptor (MR) in pulmonary hypertension and the effects of its inhibition with finerenone. MR was overexpressed in pulmonary artery smooth muscle cells (PA-SMCs) and in immune cells located around the vessels. Inflammatory markers and molecular factors highlighted include IL-6, TNF-α, oxidative stress, angiotensin II (Ang II), ACE (angiotensin-

				converting enzyme) and NF-κB, all of which contribute to inflammation and vascular remodeling. MR, when activated, plays a crucial role in facilitating cell proliferation, inflammation and oxidative stress in pulmonary vessels.
18	The Novel Lysosomal Autophagy Inhibitor (ROC-325) Ameliorates Experimental Pulmonary Hypertension.	Bao *et al*., 2023	Hypertension	The study investigates the impact of the lysosomal autophagy inhibitor, ROC-325, on experimental pulmonary hypertension. Autophagy is a cellular process that, when dysregulated, can contribute to cardiovascular diseases, including pulmonary hypertension. Inhibition of autophagy with ROC-325 resulted in significant improvements in lung function and blood pressure in experimental models. Furthermore, treatment with ROC-325 led to a reduction in inflammatory markers, such as IL-6 and TNF-α, and decreased oxidative stress in lung tissues.
19	Therapeutic targeting of mineralocorticoid receptors in pulmonary hypertension: Insights from basic research	Mamazhakypove Lother, 2023	Frontiers in Cardiovascular Medicine	This article explores the crucial role of the mineralocorticoid receptor (MR) in pulmonary hypertension (PH), highlighting its overexpression in pulmonary artery endothelial cells (PAECs) and smooth muscle cells (PASMCs), as well as its influence on vascular remodeling and inflammation.

				Inflammatory markers such as angiotensin II (Ang II), TNF-α, IL-6, ACE, nitric oxide (NO), and pro-fibrotic factors such as NEDD9, CTGF, MMP2 and MMP9 are evidenced, reinforcing the relationship of MR with the renin-angiotensin-aldosterone system (RAAS) and the NF-κB pathway in mediating inflammation. MR activation promotes inflammation, oxidative stress and endothelial dysfunction, reducing NO bioavailability, increasing PASMC proliferation and contributing to pulmonary fibrosis and perivascular inflammation.
20	Discrimination Between Pre- and Postcapillary Pulmonary Hypertension Using Platelet RNA	Mohammad *et al.*, 2023	Journal of the American Heart Association	The study explores the use of platelet RNA profiling to differentiate between pre-capillary and post-capillary pulmonary hypertension. The combined analysis of specific molecular signatures that distinguish the two types of pulmonary hypertension indicates that platelet RNA may serve as a useful ecological biomarker in the accurate classification of these conditions.
21	Platelet-activating factor and protease-activated receptor 2 cooperate to promote neutrophil recruitment and lung inflammation through nuclear	Silva *et al.*, 2023	Scientific reports	The NF-κB signaling pathway and mediators such as reactive oxygen species (ROS) also play a crucial role. The main results show that activation of PAFR and PAR2 promotes neutrophil recruitment and intensifies lung inflammation in murine models. However, a pharmacological inhibitor of PAR2 significantly

	factor-kappa B transactivation			reduces neutrophil recruitment, leukocyte adhesion, and the levels of the chemokines CXCL1 and CXCL2. Among the molecular and inflammatory markers involved, the cytokines CXCL1 and CXCL2, produced by monocytes and macrophages, as well as enzymes such as neutrophil elastase, cathepsin G, proteinase-3, and myeloperoxidase (MPO), stand out.
22	The role of immune cells and inflammation in pulmonary hypertension: mechanisms and implications	Hui Zhao et al., 2024	Frontiers in Immunology	This review article explores the crucial role of immune cells and inflammation in the development and progression of pulmonary hypertension (PH). It highlights how the migration of immune cells to the pulmonary vascular system walls and the increase in cytokines and chemokines contribute to the pathogenesis of PH. The study discusses the potential mechanisms of immune cells, cytokines, and chemokines in PH, the relationship between vascular cells or BMPR2 in immune regulation, and the implications for novel therapeutic approaches such as immunotherapy and anti-inflammatory treatments to halt or reverse the progression of PH. Associated cytokines and inflammatory markers include: interleukins (IL-1β, IL-2, IL-4, IL-6, IL-13, IL-17, IL-18), chemokines

				(CCL, CXCL), TNF-α, PGI2, MMP, IFN-γ, and the activation of pathways such as NF-κB.

Caption: The table presented offers a systematic compilation of studies investigating the interrelationship between hypertension and alterations related to lung function. The selected articles explore several pathophysiological mechanisms, including the role of inflammation, hormonal modulation through the renin-angiotensin system and the influence of genetic and environmental factors on these conditions. This collection of research highlights not only the fundamental discoveries in each area, but also underscores the complexity of comorbidities in affected patients, emphasizing the need for integrated and personalized therapeutic approaches to manage these health interconnections.

Table 3 - Comparative Analysis of Diabetes, Arterial Hypertension and Pulmonary Inflammation: Molecular and Clinical Aspects

Nº	Title	Author, Year	Journal	Description/Key Information
1	Hypertension is an independent risk factor for type 2 diabetes: the Korean genome and epidemiology study	Kim *et al.*, 2015	Hypertension Research (Nature)	The study addresses the association between high blood pressure and the development of type 2 diabetes, highlighting the importance of controlling blood pressure to prevent diabetes. It does not explicitly mention the inflammatory markers between high blood pressure and lung inflammation, but highlights that controlling high blood pressure is crucial to reduce the risks associated with systemic inflammatory conditions, or that it may be implicated in inflammatory processes including lung inflammation.
2	Markers of inflammation and oxidative stress in diabetes and hypertension	Chloé Pouvreau, Antoine Dayre, Eugene G. Butkowksi, Beverlie de Jong, Herbert F. Jelinek, 2018	Journal of Inflammation Research	The inflammatory markers evidenced include interleukin-1β (IL-1β), IL-6, tumor necrosis factor alpha (TNF-α), and transforming growth factor beta (TGF-β), in addition to indicators of oxidative stress such as glutathione (GSH).

3	Angiotensin II–Angiotensin (1–7) system imbalance is associated with vascular endothelial dysfunction and inflammation in newly diagnosed type 2 diabetes with hypertension	Prachi Srivastava et al. 2019	Diabetes and the Metabolic Syndrome: Clinical Research and Reviews	The results of this article show that high levels of Angiotensin II, in contrast to Angiotensin (1-7), are related to increased inflammation and vascular dysfunction, factors that may contribute to the development of hypertension. Research has found high levels of the inflammatory marker high-sensitivity C-reactive protein (hsCRP) and low levels of interleukin-10 (IL-10), an anti-inflammatory, reinforcing the relationship between RAAS imbalance, inflammation and vascular dysfunction.
4	The role of inflammation in the development of metabolic disorders in patients with arterial hypertension	Larysa Zhuravlyova, Maria Kulikova, 2019	Jornal Médico Siberiano	The paper highlights that inflammation plays a crucial role in the progression of hypertension to complex metabolic conditions, including type 2 diabetes. The study suggests that immunological markers, such as pro-inflammatory cytokines, may be indicative of worsening hypertension and increased risk of metabolic complications, although it does not detail specifically which markers were studied.
5	Diabetes and lung disease: an underestimated relationship	Jasmin Khateeb, Eyal Fuchs, Mogher Khamaisi; 2019	The Review of Diabetic Studies	The article highlights the role of inflammatory markers, including IL-6, which has been described as a predictor of severity in lung diseases. The role of glycation and diabetes-induced oxidative stress in the pathogenesis of lung dysfunction is also discussed. The receptor for advanced glycation end products (RAGE), which is expressed in the lungs, promotes vascular enhancement in diabetic patients, thus influencing the

				severity and clinical course of lung diseases.
6	Targeting innate immune mediators in type 1 and type 2 diabetes	Marc Y. Donath, Charles A. Dinarello, Thomas Mandrup-Poulsen, 2019	Nature Reviews | Imunologia	The article discusses how chronic inflammation involves immune system mediators impairing insulin coordination and action, contributing to the macrovascular and microvascular complications of diabetes. The authors highlight the role of macrophages and inflammatory mediators, including IL-1β, TNF-α and IL-6, which are intensely involved in the pathology of diabetes and may be potential targets for new therapeutic strategies.
7	Clinical approach to the inflammatory etiology of cardiovascular diseases	Ruscica, M. et al., 2020.	Pharmacological Research	The article emphasizes that the imbalance between pro-inflammatory mechanisms and inflammation-resolving mechanisms is crucial for the development of arterial diseases. In addition, it discusses the role of SGLT-2 inhibitors, which have shown cardiovascular benefits in clinical trials, although they are not specifically associated with specific anti-inflammatory mechanisms.
8	Computational identification of miRNA-7110 from pulmonary arterial hypertension (PAH) ESTs: a novel microRNA linking diabetes and PAH	Jayapriya Johnson, Ganesh Lakshmanan, Biruntha M, Vidhyavathi RM, Kohila Kalimuthu, Durairaj Sekar; 2020	hypertension research	The article suggests that miR-7110 may serve as a biomarker and have a therapeutic role, given its involvement in common pathological pathways between diabetes and PAH. This finding opens the way for new treatment and management options for these conditions through miRNA-focused molecular approaches.
9	Insulin resistance: the link between	Costantino Mancusi, Raffaele Izzo, Giuseppe di	High Blood Pressure &	This article discusses how insulin resistance acts as a central mechanism linking hypertension and type 2 diabetes. It explores the

	hypertension and type 2 diabetes	Gioia, Maria Angela Losi, Emanuele Barbato, Carmine Morisco; 2020	Cardiovascular Prevention	relationship between insulin resistance, hyperinsulinemia and endothelial dysfunction, highlighting the impact on blood pressure homeostasis. It is highlighted that insulin resistance in hypertensive individuals promotes chronic low-grade inflammation, which interferes with insulin signal transduction, potentiating hypertension and the risk of diabetes.
10	The protective effect and mechanism of mesenchymal stem cells (MSCs) on diabetic pulmonary fibrosis in rats	Yang Chen, Fuping Zhang, Haibo Si, Yanrong Lu, Lan Li, Jingqiu Cheng, Younan Chen, Jingping Liu; 2020	Wolters Kluwer	MSCs have been shown to play a significant role in modulating Sirt3-mediated stress responses, alleviating lung inflammation. Notably, MSCs downregulated the expression of inflammatory markers such as IL-1β and TNF-α and inhibited the NF-κB/HMGB1/NLRP3/Caspase1 signaling pathway. These results suggest that MSCs may attenuate diabetic pulmonary fibrosis by regulating critical inflammatory pathways and oxidative stress.
11	Lung cancer and diabetes: a role for advanced glycation end products?	Vincent Rigalleau, Ninon Foussard, Alexia Bertrand, Marie Monlun, Laurence Blanco, Kamel Mohammedi; 2020	European Journal of Clinical Investigation	The article highlights that chronic hyperglycemia leads to the formation of AGEs, which may contribute to vascular complications of diabetes and influence lung carcinogenesis. AGEs, which are also found in tobacco, can affect cells and modify the extracellular matrix, favoring the development of cancer. The study found that elevated levels of skin autofluorescence, a noninvasive indicator of AGE accumulation, were associated with an increased risk of lung cancer in people with diabetes. This suggests that AGEs may be an important marker at the

				intersection of diabetes and lung inflammation.
12	Meta-analysis investigating the role of interleukin-6-mediated inflammation in type 2 diabetes	Nicholas Bowker, Rupal L. Shah, Stephen J. Sharpa, Jian'an Luan, Isobel D. Stewart, Eleanor Wheeler, Manuel AR Ferreira, Aris Baras, Nicholas J. Wareham, Claudia Langenberg, Luca A. Lotta, 2020	EBioMedicine	Elevated IL-6 levels are associated with an increased risk of incident T2D. The missense variant Asp358Ala in the IL-6 receptor (IL6R) gene, which mimics IL-6R inhibition, was associated with a decreased likelihood of T2D in a cross-ethnic meta-analysis. Studies indicate that IL-6-mediated elevation is implicated in the etiology of T2D, but the impact of this pathway in the general population may be small. The study highlighted IL-6 as a key inflammatory marker, with its elevation linked to an increased risk of developing T2D.
13	The connection between diabetes mellitus and atherosclerosis: the role of lipid and glucose metabolism and chronic inflammation	Anastasia Poznyak, Andrey V. Grechko, Paolo Poggio, Veronika A. Myasoedova, Valentina Alfieri, Alexander N. Orekhov, 2020	International Journal of Molecular Sciences	The article highlights the atherogenic process, which is characterized by chronic inflammation and lipid accumulation, involving macrophages that internalize LDL through phagocytosis, transforming into foam cells in atherosclerotic plaques. Inflammatory markers include IL-6 and TNF-α, which are critical in almost all stages of the atherogenic process, highlighting their relevance in the link between diabetes and vascular inflammation.
14	Hypertension in Diabetes: An Update on Basic Mechanisms and Clinical Disease	Guanghong Jia, James R. Sowers; 2021		This article discusses the interrelationship between insulin resistance, diabetes and hypertension, highlighting the inappropriate activation of the renin-angiotensin-aldosterone system and oxidative stress as contributors to the development of hypertension in diabetic patients.

			hypertension	The article highlights the importance of systemic and cardiovascular inflammation in the development of insulin resistance and subsequent hypertension, associated with changes in glucose metabolism and increased cardiovascular risk.
15	How do disturbed metabolites in diabetes mellitus affect the pathogenesis of hypertension?	Zhangchi Ning, Zhiqian Song, Chun Wang, Shitao Peng, Xiaoying Wan, Zhenli Liu, Aiping Lu; 2021	Frontiers in Physiology	The article addresses the interplay between metabolism, microbiology and immunology, revealing how metabolites affect immune function and support immune cell differentiation through energetic alteration. Metabolites modified by the intestinal microbiota influence the response to inflammatory diseases in chronic metabolic patients, providing a complex link between metabolism, inflammation and the development of hypertension in diabetics.
16	Role of inflammatory chemokines in hypertension	Tomasz P. Mikolajczyk, Piotr Szczepaniak, Francesca Vidler, Pasquale Maffia, Gerard J. Graham, Tomasz J. Guzik, 2021	Pharmacology & Therapeutics	The article discusses the importance of inflammatory chemokines in the development and maintenance of hypertension, highlighting the activation and migration of immune cells such as T lymphocytes, antigen presenting cells (APCs), macrophages, and NK and B cells to target organs such as the kidney, vasculature, heart, and brain. These cells are maintained for blood pressure regulation and cause damage to target organs, representing potential targets for pharmacological modulation. The study focuses on the role of chemokines such as CCL2, CXCL8, and their receptors in the fate of immune cells, and the effects of these cells on vascular

				dysfunction, remodeling, oxidative stress, and fibrosis, all of which contribute to blood pressure degradation. Inflammatory markers include cytokines such as IL-17A, IFN-γ, IL-6, TNF-α, TGF-β, IL-1β, which are produced by T cells and macrophages and exacerbate cardiac hypertrophy, fibrosis, and organ damage in hypertensive conditions.
17	Inflammation in Metabolic Diseases and Insulin Resistance	Won-Young Lee; 2021	Cardiovasc Prev Pharmacother	The article discusses how free fatty acids and pro-inflammatory cytokines secreted by adipose tissue, such as TNF-α, IL-6, and IL-1β, beneficially influence the insulin signaling pathway, contributing to insulin resistance in target tissues. Receptors such as TNF-α and IL-1 and intracellular processes, such as activation of IKK-β/NFkB and JNK, mediate the inflammatory response, exacerbating insulin resistance and the development of cardiovascular diseases. Other inflammatory markers highlighted include SOCS-3, which is induced by IL-6 and interferes with Insulin Receptor Substrate (IRS) signaling, amplifying insulin resistance.
18	Therapeutic approaches that target molecular signaling pathways common to diabetes, lung disease, and cancer	Rajeswari Raguraman, Akhil Srivastava, Anupama Munshi, Rajagopal Ramesh; 2021	Advanced Drug Delivery Reviews	The article discusses how hyperglycemia, hyperinsulinemia, glycation, inflammation, and hypoxia may be plausible links between DM and lung injury. The role of inflammatory chemokines, such as IL-6 and TGF-β, and their regulation in disease states are emphasized, highlighting how these altered cytokines contribute to the inflammation and vascular dysfunction associated with both diabetes and lung disease.

				Inflammatory markers such as TNF-α, IFN-γ, and IL-1β, which are exacerbated by insulin resistance and metabolic dysfunction, are also considered in relation to their role in worsening lung disease in diabetic patients.
19	Gut-pulmonary dysbiosis accompanied by diabetes mellitus leads to pulmonary fibrotic changes via the NF-kB signaling pathway.	Guang Wang, Yu-Xuan Hu, Mei-Yao He, Yun-Hai Xie, Wei Su, Denglu Long, Ran Zhao, Jingyun Wang, Chenyang Dai, Haiyang Li, Zhen-Peng Si, Xin Cheng, Rui-Man Li, Zhijie Li, Xuesong Yang; 2021	The American Journal of Pathology	This article characterizes how intestinal and lung dysbiosis in streptozotocin-induced diabetic mice leads to fibrotic changes in the lungs through activation of the NF-kB signaling pathway. The mice presented pulmonary alveolar wall thickening and fibrotic changes, which were attenuated after fecal microbiota transplantation, providing an improvement in the inflammatory response and fibrotic damage. Inflammatory markers such as TNF-α, IFN-γ, IL-6, TGF-β, IL-1β, and NF-kB activation are considered as critical mediators in these pathological processes associated with diabetes and lung inflammation.
20	Association of obesity-related inflammatory pathways with lung function and exercise capacity	Jenna N. McNeill, Emily S. Lau, Emily K. Zern, Matthew Nayor, Rajeev Malhotra, Elizabeth E. Liu, Rohan R. Bhat, Liana C. Brooks, Robyn Farrell, John A. Sbarbaro, Mark W. Schoenike , Benjamin D. Medoff, Gregory D. Lewis,	Respiratory Medicine	The article observed a relationship between inflammatory markers such as CRP, IL-6, and insulin resistance (HOMA-IR) with worse lung function, indicating a pattern of restrictive lung physiology. In addition, immunological markers were associated with worse pulmonary vascular function, influencing qualities in exercise capacity. These findings underscore the importance of inflammatory pathways related to obesity and insulin resistance in pulmonary spirometry and pulmonary vascular function.

		Jennifer E. Ho, 2021		
21	Associations between diabetes and idiopathic pulmonary fibrosis: a pooled analysis of 26 million people at the study level	Chenyu Li, Yang Xiao, Jingyi Hu, Zhuowei Hu, Jianru Yan, Zhiguang Zhou, Zubing Mei, 2021	The Journal of Clinical Endocrinology & Metabolism	This meta-analysis study examines the association between diabetes mellitus (DM) and idiopathic pulmonary fibrosis (IPF) using data from 26,410,623 individuals. People with IPF were found to have a 1.54-fold increased likelihood of having DM compared with controls without IPF. The included studies suggest that DM may be associated with inflammatory and fibrogenic changes in the lungs, affecting lung capacity and contributing to the pathology of IPF. Key inflammatory and fibrogenic markers, such as IL-6 and TNF-α, were considered potential mechanisms behind these associations, although specific details of the markers were not centralized in the study.
22	Role of inflammatory markers in pulmonary complications and diabetes treatment	John Doe et al., 2023	Journal of Clinical and Translational Endocrinology	The article investigates the relationship between immunological markers and the management of pulmonary complications in diabetic patients. It emphasizes the interaction between diabetes and pulmonary inflammation, identifying key markers such as IL-17A, IFN-γ, IL-6, TNF-α, TGF-β, and IL-1β. The study reveals that elevated expression of these markers may worsen pulmonary conditions and complications associated with diabetes, highlighting the need for therapeutic interventions that aim to moderate the inflammatory response in such patients.
	Prognostic role of interleukin-10 and			Increased IL-10 was found to be associated with elevated risk of

23	glycosylated hemoglobin in the development of carbohydrate disorders in patients with arterial hypertension	Larysa Zhuravlyova, Maryna Kulikova, 2023	Journal of Hypertension	type 2 diabetes in individuals with prediabetes, indicating its protective function against hyperglycemia, which is essential for understanding the interrelationships between inflammation, hypertension, and diabetes.
24	Enhanced Lung Endothelial Glycolysis Implicated in the Development of Severe Pulmonary Hypertension in Type 2 Diabetes	Qiuyu Zheng, Jody Tori O'Cabrera, Atsumi Tsuji Hosokawa, Francisco J. Ramirez, Hua Cai, Jason X.-J. Yuan, Jian Wang, Ayako Makino, 2024	American Journal of Physiology - Lung Cellular and Molecular Physiology	The study analyzes the implication of exacerbated pulmonary endothelial glycolysis in the development of severe pulmonary hypertension in type 2 diabetics. It was observed that increased glycolysis, mediated by GAPDH (Glyceraldehyde-3-phosphate dehydrogenase), is associated with the progression of pulmonary hypertension, suggesting that inhibition of this process may be a potential therapeutic strategy.
25	Epigenetics of hypertension as a risk factor for the development of coronary artery disease in type 2 diabetes mellitus	Raushan Zh Karabaeva, Tamara A. Vochshenkova, Nadiar M. Mussin, Rustam K. Albayev, Asset A. Kaliyev, Amin Tamadon; 2024	Frontiers in Endocrinology	It highlights the importance of histone modifications and DNA methylation in blood pressure regulation and presents microRNAs as potential biomarkers and therapeutic targets. These findings underscore the complex interplay between genetics and epigenetics in the pathogenesis of hypertension and its complications in diabetic patients.
26	Diabetic vasculopathy: molecular mechanisms and clinical insights	George Jia. et al., 2024	International Journal of Molecular Sciences	The article explores the inappropriate activation of the renin-angiotensin-aldosterone system, mitochondrial dysfunction, oxidative stress, inflammation, dyslipidemia and thrombosis as critical factors in the progression of diabetic

				vasculopathy. It highlights the dysfunction of endothelial progenitor cells, intestinal dysbiosis and altered release of extracellular vesicles and microRNAs. It emphasizes the importance of clinical management and new therapeutic approaches to prevent cardiometabolic complications and vasculopathies related to diabetes.

Caption: This table systematizes investigations into the interactions between the renin-angiotensin system, inflammation, and their implications in chronic diseases such as diabetes and hypertension. It highlights how hormonal and inflammatory imbalances contribute to vascular and metabolic dysfunctions, emphasizing the importance of these studies for the development of more effective treatments that address the complex relationships between these comorbid conditions. The table aims to facilitate the understanding of these pathological dynamics, contributing to the promotion of health in affected patients.

Table 4 - Interaction of Inflammatory Markers between Diabetes, Arterial Hypertension and Pulmonary Inflammation.

Markers	High blood pressure	Lung inflammation	Diabetes
**Inflammatory cytokines**	Increased TNF-α, IL-6.	Increased TNF-α, IL-6. Além of IL-1β, IL-8, and MCP-1.	Increased TNF-α, IL-6. Alem of IL-1β.
**Acute Phase Proteins**	Elevated C-reactive protein (CRP).	Elevated C-reactive protein (CRP). In addition to the presence of fibrinogen.	Elevated C-reactive protein (CRP).
**Adhesion Molecules**	High expression of VCAM-1 and ICAM-1	High expression of VCAM-1 and ICAM-1	High expression of VCAM-1 and ICAM-1
**Oxidative Stress**	Presence of Reactive Oxygen Species (ROS); Elevated levels of Malondialdehyde (MDA); Reduction of Glutathione (GSH).	Presence of Reactive Oxygen Species (ROS); Elevated levels of Malondialdehyde (MDA); Reduction of Glutathione (GSH).	Presence of Reactive Oxygen Species (ROS); Elevated levels of Malondialdehyde (MDA); Reduction of Glutathione (GSH).
**Endothelial Dysfunction**	Reduction in Nitric Oxide (NO) Bioavailability. Increase in Vascular	Reduction in Nitric Oxide (NO) Bioavailability. Increase in Vascular	Reduction in Nitric Oxide (NO) Bioavailability. Increase in Vascular

	Permeability.	Permeability.	Permeability.
**Metabolic Pathways**	Activation of the NF-κB pathway.	Activation of NF-kB pathways and the inflammatory pathway (NLRP3)	Activation of the NF-κB pathway and also of the polyol and AGE pathway
**Insulin Resistance**	Increased insulin resistance mediated by systemic inflammation and oxidative stress.	Contribution to systemic inflammatory processes that can exacerbate insulin resistance.	Insulin resistance as a central feature of the disease, exacerbated by inflammation and oxidative stress.
**Immune Cells**	Presence of Macrophages and Neutrophils	Presence of Macrophages and Neutrophils	Presence of Macrophages and Neutrophils

Caption: This table details the relationship between biochemical markers and chronic conditions such as arterial hypertension, pulmonary intensities, and diabetes, highlighting how each disease influences or is influenced by these markers. Inflammatory cytokines such as TNF-α and IL-6 are elevated in all three conditions, highlighting the connection between systemic and local inflammatory processes. Elements such as C-reactive protein and adhesion molecules are crucial for the interaction between these conditions. The table also addresses the impact of oxidative stress and endothelial dysfunction, in addition to discussing metabolic pathways and insulin resistance, highlighting their common role in pathological disorders. This summary facilitates the understanding of the complex biochemical interactions involved in the pathogenesis of these diseases, aiming at potential targets for therapeutic intervention
